# Associations between bride price stress and intimate partner violence amongst pregnant women in Timor-Leste

**DOI:** 10.1186/s12992-017-0291-z

**Published:** 2017-08-28

**Authors:** Susan Rees, Mohammed Mohsin, Alvin Kuowei Tay, Elisa Soares, Natalino Tam, Zelia da Costa, Wietse Tol, Derrick Silove

**Affiliations:** 1 Psychiatry Research and Teaching Unit, Academic Mental Health Unit, School of Psychiatry, University of New South Wales, Cnr Forbes and Campbell Streets, Liverpool, NSW 2170 Australia; 2 Johns Hopkins University, Baltimore, USA; 3 Sydney, NSW 2052 Australia

**Keywords:** Stress, Bride price, Poverty, Intimate partner violence

## Abstract

**Background:**

Reducing violence against women is a global public health priority, particularly in low-income and conflict-affected societies. However, more needs to be known about the causes of intimate partner violence (IPV) in these settings, including the stress of bride price obligations.

**Methods:**

The representative study of women attending ante-natal clinics in Dili, Timor-Leste was conducted between June, 2013 and September, 2014 with 1672 pregnant women, a response rate of 96%. We applied contextually developed measures for the stress of bride price and poverty, and the World Health Organisation measure for intimate partner violence.

**Results:**

Compared to those with no problems with bride price, women with moderate or serious problems with that custom reported higher rates of IPV (18.0% vs. 43.6%). Adjusting for socio-demographic factors, multivariate analysis revealed that ongoing poverty (OR = 1.75, 95% CI: 1.20–2.56) was significantly associated with IPV. Importantly, the strongest association with IPV was problems with bride price (OR = 2.73, 95% CI: 1.86–4.01).

**Conclusions:**

This is the first large consecutively sampled study to demonstrate a strong association between the stressors of bride price and poverty with IPV. Notably, bride price stress had the strongest association with IPV. Revealing this hitherto unrecognized factor of bride price stress may prove pivotal in guiding policy and interventions aimed at reducing IPV, and thereby improve the health and psychosocial status of women in low income and conflict-affected settings.

**Electronic supplementary material:**

The online version of this article (doi:10.1186/s12992-017-0291-z) contains supplementary material, which is available to authorized users.

## Background

One of the greatest public health challenges of our time is to assist countries affected by the dual problems of extreme poverty and exposure to mass conflict to achieve sustainable peace and economic advancement [1]. The pivotal role of women in achieving these goals has been emphasized, but their capacity to participate fully in development is compromised by a range of risk factors affecting their health and well-being, including exposure to intimate partner violence (IPV) [2].

Evidence is conclusive that IPV represents a pervasive risk factor for adverse physical and mental health outcomes amongst women in low income, conflict-affected countries [3]. The scale of harm caused to women has elevated IPV to a central focus in the Sustainable Development Goals (SDGs). Several factors are known to converge in contributing to the high rates of IPV in low-income countries including gender inequality, societal tolerance and supportive attitudes of violence against women, poverty, male underemployment, low education in women, childhood exposure to abuse, and male alcohol and drug use. [4, 5] Yet, given the pervasive nature of these risk factors, major questions remain as to why some men resort to violence whereas others do not.

It is remarkable that no epidemiological studies have considered the possibility that bride price, and the stress it causes within families, especially if already affected by poverty, may contribute to increased risk of violence against women. It is possible that a failure to understand or respond to this hidden contributor to IPV may limit the effectiveness of programs aimed at curtailing it.

Our focus on pregnant women was influenced by several considerations. In general, younger adult women are known to be at increased risk of IPV [4–6]. Within that socio-economic grouping, the child-bearing phase may accentuate the financial pressures of bride price both because of an increase in traditional ceremonial costs associated with childbirth and the reduced economic capacity of women in the antenatal period. Together, these factors may contribute to the overall economic pressures on families, with ensuing stress and resort to blame increasing the risk of IPV. For these reasons, our study focuses on assessing associations between the stresses of bride price and IPV, taking into account the role of poverty, in a low-income, post-conflict country, Timor-Leste.

### Bride price

Although the custom of bride price varies in its detail and implementation across diverse cultures, the core universal element involves the transfer of offerings, goods or funds principally from the groom and his family, to the bride’s family [7]. Bride price obligations occur both at the time of marriage, and in many societies including Timor-Leste, for an indefinite period thereafter [8, 9]. Commonly, increased expectations of payments and offerings occur during ceremonial events including the birth of children, the prospect resulting in the perinatal period being a period of high stress for the couple [9, 10]. It is important to distinguish bride price from the practice of dowry in which the transfer is from the bride’s family to the new couple. It is notable however, that even where the practice involves the reverse exchange of goods and funds, such as in India and Pakistan, the practice is associated with violence against women [11–13].

The bride price custom is widespread, being practiced in countries throughout Asia, Africa, the Middle East and the Pacific, and extending to women from these regions who are immigrant and refugees resettled or seeking asylum in high income countries. Although in aggregate, the at risk population exceeds two billion women, the exact number affected by stress related to bride price remains to be determined (see Table 1). There are reasons to hypothesize that the bride price custom may put women at risk in relation to their overall gender status and, more specifically, to IPV. Qualitative data offer support for these associations [9, 14–17]. In a cross sectional study of pregnant women in Timor-Leste, we found an associations of bride price stress with impairment in women’s mental health, and more generally, with increased family and spousal conflict. The design of the study did not allow, however, for a direct examination of a possible relationship between bride price and IPV [8]. Notably, a cross-sectional multi-country study found inconclusive results when examining for associations between the customs of bride price and dowry and risk of IPV. Although the custom of dowry was documented in six sites only four had higher than average levels of IPV and one had lower IPV rates. Bride price was associated with decreased IPV in four sites and increased IPV in two sites [18].

**Table 1:** Bride Price

Country where bride price is practiced	Population (total)	Population (women)	Poverty index
Bride Price (Net assets move from groom’s family to bride’s family) by Country and Poverty Index by Country			
Afghanistan	31,411,743	15,160,172	15.8
Algeria	35,468,208	17,567,864	22.6
Angola	19,081,912	9,632,075	36.6
Bahrain	1,261,835	473,999	N/A
Bangladesh	148,692,131	73,383,331	22
Botswana	2,006,945	995,092	19.3
Brunei	398,920	197,358	N/A
Burkina Faso	16,468,714	8,296,443	9.7
Burundi	8,382,849	4,269,467	66.9
Cambodia	14,138,255	7,221,673	30.1
Cameroon	19,598,889	9,816,338	39.9
Central African Republic	4,401,051	2,233,189	62.0
Chad	11,227,208	5,644,877	46.7
China	1,341,335,152	644,994,400	N/A
Democratic Rep. of the Congo	65,965,795	33,157,496	71.3
Republic of the Congo	4,042,899	2,019,306	46.5
Djibouti	888,716	444,166	N/A
East Timor	1,124,355	551,191	49.9
Egypt	81,121,077	40,387,475	25.2
Equatorial Guinea	700,401	341,408	76.8
Eritrea	5,253,676	2,666,033	69.0
Ethiopia	82,949,541	41,667,964	38.9
Gabon	1,505,463	750,454	32.7
Gambia	1,728,394	874,877	48.4
Guinea	9,981,590	4,937,969	55.2
Guinea-Bissau	1,515,224	764,258	69.3
Hong Kong	7,053,189	3,711,339	N/A
India	1,224,614,327	592,067,546	22.0
Indonesia	239,870,937	120,248,498	12.5
Iran	73,973,630	36,432,408	N/A
Iraq	31,671,591	15,784,742	22.9
Ivory Coast (Cote D’Ivoire)	19,737,800	9,680,280	42.7
Jordan	6,187,227	3,004,996	14.4
Kazakhstan	16,026,367	8,331,739	8.2
Kenya	40,512,682	20,278,981	45.9
Kuwait	2,736,732	1,103,460	N/A
Kyrgyzstan	5,334,223	2,702,477	33.7
Laos	6,200,894	3,107,050	27.6
Lebanon	4,227,597	2,163,457	N/A
Lesotho	2,171,318	1,104,192	56.6
Liberia	3,994,122	1,987,469	63.8
Libya	6,355,112	3,132,342	N/A
Madagascar	20,713,819	10,388,280	75.3
Malawi	14,900,841	7,446,051	50.7
Malaysia	28,401,017	13,993,650	3.8
Mali	15,369,809	7,691,028	43.6
Mauritania	3,459,773	1,721,146	42
Morocco	32,481,912	16,532,592	9
Mozambique	23,390,765	12,010,341	54.7
Myanmar	47,963,012	24,323,127	N/A
Namibia	2,283,289	1,149,298	28.7
Nepal	29,959,364	15,097,145	25.2
Niger	15,511,953	7,710,241	59.5
Nigeria	158,423,182	78,222,179	46
Oman	2,782,435	1,148,710	N/A
Pakistan	173,593,383	85,356,405	17.2
Papua New Guinea	6,858,266	3,359,979	39.9
Qatar	1,758,793	427,853	N/A
Rwanda	10,624,005	5,408,996	44.9
Saudi Arabia	27,448,086	12,251,954	N/A
Senegal	12,433,728	6,266,846	46.7
Sierra Leone	5,867,536	3,001,444	52.9
Singapore	5,086,418	2,521,975	N/A
Solomon Islands	538,148	259,909	3.2
Somalia	9,330,872	4,703,761	N/A
South Africa	50,132,817	25,308,517	23
South Sudan	13,024,734	6,462,444	50.6
Sri Lanka	20,859,949	10,560,835	8.9
Sudan	43,551,941	21,609,210	46.5
Swaziland	1,186,056	603,061	63
Syria	20,410,606	10,080,745	N/A
Taiwan	23,216,236	11,468,433	N/A
Tajikistan	6,878,637	3,493,864	46.7
Tanzania	44,841,226	22,443,881	28.2
Thailand	69,122,234	35,149,886	13.2
Togo	6,027,798	3,042,605	58.7
Trinidad and Tobago	1,341,465	691,380	N/A
Tunisia	10,480,934	5,240,866	15.5
Turkey	72,752,325	36,467,075	18.1
Turkmenistan	5,041,995	2,559,364	N/A
Turks and Caicos Islands	36,196	17,174	N/A
Ganda	33,424,683	16,721,224	24.5
United Arab Emirates	7,511,690	2,288,096	N/A
Uzbekistan	27,444,702	13,803,958	16
Vanuatu	239,651	117,573	N/A
Vietnam	87,848,445	44,430,545	17.2
Yemen	24,052,514	11,948,920	8.9
Zambia	13,088,570	6,529,515	59.3
Zimbabwe	12,571,454	6,374,259	72.3
Total	4,644,257,765	2,184,975,205	

**Sources & References**

UN Statistics Division, Department of Economic and Social Affairs. “World Population Prospects: The 2010 Revision”. www.un.org/en/development/desa/population/publications/pdf/trends/

World Bank Poverty Index: https://en.wikipedia.org/wiki/List_of_countries_by_percentage_of_population_living_in_poverty http://data.worldbank.org/indicator/SI.POV.NAGP

“South Sudan population literacy” is based on the latest data published by UNESCO Institute for Statistics (retrieved March 13, 2016)

The estimation data for section “Turks and Caicos Islands age structure” is based on the latest demographic and social statistics by United Nations Statistics Division **N/A:** Not Available

Our study is grounded in both Feminist and Marxist theory. The assumption is that assigning a cost to a woman inevitably ‘commodifies’ her as an object of transaction in all cultures. This financial value placed on women has manifold effects, for example, increasing the risk that daughters will be offered for early marriage, a practice that undermines the potential for a gender equal marital relationship, and which likely increases the risk of violence against the women enacted by the husband or his family [17, 19, 20]. Allocating a price to a woman also legitimizes the right and power of men to continue to treat their female partner as an acquired object. As such, the custom of bride price may maintain and strengthen other known risk factors for IPV, including community attitudes and perceptions that support gender inequality and male entitlement to treat women violently [21].

### Conflict and poverty

Timor-Leste has experienced a prolonged history of conflict, the massive disruption to the society impacting adversely on pervasive conditions of poverty and underdevelopment [1]. The country was occupied by Indonesia in 1975, provoking a protracted resistance war which culminated in a humanitarian emergency in 1999. Approximately 600,000 persons died. There was loss of property and livelihoods [22], and the ﻿country is ranked as one of the poorest in the world [23].

As in other low-income, post-conflict countries, gender inequality is pervasive in Timor-Leste, with women being disproportionately disadvantaged in all walks of life [2, 20–25]. Close to half of women interviewed in a national survey in the capital city (Dili) and surrounding district reported exposure to IPV, a figure that is comparable to other low-income, conflict-affected countries [24, 25]. High fertility rates take a major toll on women, the maternal death rate remaining high (215 per 100,000 live births in 2015) [26].

It has been argued that, within a social system characterised by gender inequality, the stress of poverty may trigger male perpetrated IPV [4, 27]. Research also indicates that economic inequality between partners significantly heightens the risk of IPV [27]. It seems plausible therefore, that the risk of IPV may be heightened by pressures to remit the bride price. Hence, it is vital to consider the role of poverty when examining the relationship between bride price and IPV in a low-income society.

In the present analysis, we tested the following hypotheses [1]: That the stress of poverty reported by pregnant women would be associated with IPV [2]; That the stress of bride price would be associated with IPV [3]; That, taking into account sociodemographic, pregnancy-related factors and poverty, the stress of bride price would make an independent and strong contribution to IPV.

## Methods

### Study setting and sample

The DILI birth cohort study is an ongoing multi-wave inquiry conducted in the geographical district including and surrounding the capital city of Timor-Leste as described previously [28]. The baseline data reported here were obtained between June, 2013 and June, 2014 from pregnant women attending the four largest community health centres, situated in the capital city suburbs and providing antenatal care for the entire district including urban and rural areas. In the latest census, Dili district was home to 21.9% of the Timorese population of 1.1 million persons. Government statistics indicated that 96% of pregnant women in the Dili District attended antenatal clinics [24]. Only a small number of women (estimated to be less than 5%) attend smaller satellite antenatal clinics or private facilities.

The second trimester was selected because most women initiate antenatal clinic attendance during that period of pregnancy. All consecutive women were approached while registering their visits. We excluded women with psychosis, severe intellectual disability or severe medical conditions requiring referral to the national hospital in Dili. Informed consent procedures were undertaken in private. Once consent was obtained, women were asked about their preferences for an interview at the health centre or at a subsequent home visit (4.1% of interviews).

Data from a pilot study (*n* = 427) indicated that 400 participants were required to generate stable prevalence estimates for each clinic [29].

We conducted interviews with 1672 pregnant women, with a response rate of 96%.

### Data management

Surveys were checked for omissions or out of range values immediately following interview, and again at the research office prior to being entered in de-identified form by a trained data assistant into a password protected computer. Further systematic checking was undertaken of electronic files. Two records were excluded from the analysis because of missing data (>1 item). Surveys were locked in the research office prior to being transferred to the University of New South Wales in Sydney for secure storage.

### Measures

We selected measures that were theoretically and contextually relevant, had utility in a resource-poor research setting, and showed good psychometric properties based on earlier qualitative and (longitudinal) psychiatric epidemiological research with women in the Dili district [8, 29].

Bride price is known as ‘lia’ or ‘barlake’ in Timor-Leste [9]. Lia involves an obligation on the husband and his wife, once married, to provide for all traditional obligations including funerals and weddings as well as ongoing support to the female partner’s family as a form of marriage expense. In this study we included lia as an item: “not enough money for traditional obligations (lia).” The item was one of a number in a measure of self-reported daily stressors that had occurred in the last 12 months. The rating options were: no problem; some problem; moderately serious problem and serious problem (rated 1–4).

Poverty was measured by three items that were also included in the daily stressors inventory, relating to the past 12 months. Poverty items included: not enough food; poor shelter; and not enough money for school fees or clothes. Each poverty item was scored on the same scale as bride price stress. The total poverty score reflected the sum of all three item scores (ranging from 3 to 12) and following examination of the distribution of scores, the poverty index was divided into three categories: no problem, some problem, and moderately/serious problem.

IPV was assessed using the World Health Organization Violence Against Women Instrument (VAWI) [25, 30]. The timeframe was 12-month prevalence enacted by the current or most recent partner. We excluded the sexual abuse items because the topic was highly sensitive for women in a traditional and deeply religious society. Based on considerations of the distribution and overlap of the psychological and physical domains of IPV, we assigned women to five hierarchically ordered categories. The categories were identified and tested in this setting and category 5 which includes the combination of severe psychological and physical abuse was the most strongly associated with symptoms of depression and mental distress [31, 32]. The categories are [1]: No abuse (no IPV items endorsed) [2]; Low respect/regard (whether spouse spends free time with the respondent, consults her on household matters; respects her and her wishes; trusts her with money) [3]; Severe psychological abuse (jealous or angry if she talks to other men; accusations of being unfaithful; does not permit meetings with friends; limits contact with family; insists on knowing woman’s whereabouts; humiliates her; threatens harm to her or someone close; lack of affection) [4]; Physical abuse alone (pushing, shaking or throwing items; slapping, twisting arm; punching; kicking/dragging; strangling/burning; threats with knife, gun or other weapon; attacks with a knife, gun or weapon; other forms of physical abuse); and [5] Combination of physical abuse and severe psychological abuse. The typology incorporates knowledge that forms of IPV often co-occur. The typology does not assume that allocation to one category will prevent future transference to another category. Assignment of women to the higher level categories [3–5] was not dependent on whether or not they also endorsed items from category 1 [7].

### Funding

Funding was from the National Health and Medical Research Council of Australia.

### Ethics, consent and permissions

The Human Research Ethics Committee of the University of New South Wales (UNSW) and the National Health Research Cabinet of Timor-Leste approved the study (HC13049). Women were informed verbally about the study because of low literacy levels. They signed translated consent forms in the presence of a witness.

### Statistical analyses

We applied bivariate and multivariate analyses to examine the relationship between poverty, stress of bride price, and IPV. Descriptive statistics for all variables are presented followed by bivariate analyses examining associations with IPV for each of the explanatory variables including relevant sociodemographic factors, stress of bride price, stress of poverty, and pregnancy-related factors. Preliminary bivariate analysis found a statistically significant association between poverty and bride price. This finding provided the rationale for a multivariate analysis to be undertaken to assess the unique contributions of each of the relevant explanatory variables, taking into consideration the degree of co-linearity between these variables. Hence, multiple logistic regression analysis was used to explore the independent contributions of each potential explanatory variable on IPV, adjusting for other variables. Given that in preliminary analyses significant associations were found between categories 4 and 5 IPV categories, that variable was dichotomised as follows: scores of 1 to 3 = no IPV (score = 0); and 4 or 5 = physical and severe psychological abuse =1. In addition, in preliminary analyses, an association was found between parity (whether first or subsequent births) and whether pregnancies were planned or not, so that only the parity variable was included as a proxy index of both indicators in the multiple logistic regression model.

The results of bivariate analyses are expressed as percentages and chi-square tests. The results of the multiple logistic regression analysis are reported as adjusted odds ratios (OR) with 95% confidence intervals (95% CI), statistical differences being set at *p* < 0.01 and *p* < 0.05. The analyses were performed using IBM SPSS version 22.

## Results

The socio-demographic characteristics of the sample of women are presented in Table 2. Around two-thirds (68.8%) of participants were between 20 and 29 years of age, a quarter (25.1%) were married and 74.2% were engaged or living together (common in urban areas, while couples prepare financially for marriage). More than half the women had completed junior or senior high school and one fifth (20.1%) held a university degree. Nevertheless, more than half (56.8%) were unemployed.

**Table 2:** Socio-Demographic Characteristics of Sample and Bride Price Stress, Poverty Stress and Intimate-Partner Violence (IPV)

Characteristics	Number of women (*n* = 1672)	Col %
Age groups		
Younger than 20 years	141	8.4
20–24	568	34.0
25–29	576	34.4
30–34	273	16.3
35 and above	114	6.8
Marital status		
Married	419	25.1
Engaged/living together	1240	74.2
Separated/divorced	9	0.7
Educational status		
None or Primary school	271	16.2
Junior/Senior High School	961	57.5
Technical College/Diploma	104	6.4
University	336	20.1
Occupation		
Unemployed	949	56.8
Student	180	10.8
Farming/small trade	200	12.0
Government/Non-Government Organisation (NGO)	190	11.4
Other	153	9.2
Bride price stress		
No problem at all (ref. category)	899	53.8
A bit of a problem	600	35.9
Moderate/ serious/very serious problem	173	10.3
Ongoing poverty-related stress^1^		
No problem at all (ref. category)	802	48.0
Some problem	668	40.0
Moderately serious /very serious problem	202	12.0
^§^Intimate-partner violence (IPV) derived groups#		
No abuse at all	180	10.8
Low respect/regard and no abuse	550	32.9
Severe psychological abuse (threatening, intimidation and controlling)	511	30.6
Physical abuse only	103	6.2
Physical abuse + Severe psychological abuse (threatening, intimidating and controlling)	327	19.5

^1^Poverty stress included following three items (each item scored 1 to 4: 1. not a problem 2. a bit of problem 3. moderately serious problem 4. a serious problem; total score ranges from 3 to 12): Not enough food; Poor shelter; Not enough money for school fees

^§^Intimate-partner violence (IPV) items are grouped as follows

Low respect/regard only: Participants included if endorsed one or more items from this group but not from forms of IPV categorized higher in the hierarchy. Example of items include spends his free time with you; consults on different household matters with you; respects you and your wishes; does not trust you with any money

Severe psychological (threatening, intimidating and controlling): Participants included if endorsed one or more items from this group. Participants may have endorsed items from Low respect/regard but not included if endorsed any physical abuse items. Items included in this group are: he is affectionate with you; jealous or angry if you talk to other men; frequently accuses you of being unfaithful; does not permit you to meet your girl friends; tries to limit your contact with your family; insists on knowing where you are at all time; humiliates you in front of others; threatens you/someone close to you with harm

Physical violence: Participants were included if they endorsed one or more of: pushes you, shakes you or throws something at you; slaps you or twists your arm; punches you with something that could hurt you; kicks/drags you; tries to strangle/burn you; threatens you with a knife, gun, or other type of weapon; attacks you with a knife, gun, or other type of weapon; other ways your husband hurts you. Participants were included in the Physical violence only group if they did not endorse any items from the Severe psychological group. They may have endorsed items from the Low respect/ regard category

Physical violence plus Severe psychological (threatening, intimidating and controlling): Participants were included in this group if they endorsed a minimum of one item from physical violence and one from threatening/jealous/controlling. In addition, they may have endorsed items from Low respect/ regard category

#There was one missing case across all IPV items and that has been excluded from the further analyses (*n* = 1671)

Most women originated from the Dili district, but a minority had migrated to the city from all of the 13 districts of the country. More than a third (38.0%, *n* = 638) were primiparous and in 14% of the whole sample, the pregnancy was unplanned.

More than half (53.8%) of the women reported no problem with the financial stress of bride price, 35.9% experienced a bit of problem and 10.3% reported that bride price stress was a moderate or serious problem. Less than half (48.0%) reported no poverty, 40.0% endorsed having some level of ongoing poverty and 12% moderate or serious poverty (Table 2).

Table 2 shows that among the full sample of 1672 women, 180 (10.8%) reported no IPV, a third (*n* = 550; 32.9%) exposure to low regard/respect alone; 30.6% (*n* = 511) severe psychological abuse; 6.2% (*n* = 103) physical abuse; and 19.5% (*n* = 327) a combination of severe psychological and physical abuse.

We examined the bivariate relationships of socio-demographic characteristics and IPV. The rate of IPV was higher among adolescent women (29.8%) (under 20 years) as compared to older women (35 years and above) (19.8%).

Table 3 shows a regular dose–response relationship between the severity of stress of poverty and the derived IPV categories (*p* < 0.01). Women reporting moderate or serious problems with poverty had almost double the risk of IPV when compared to those with no problems (37.6% vs. 18.2%). The findings also revealed a dose–response﻿ relationship between the individual items of poverty-related stress with bride price stress (Additional file 1).

**Table 3:** Multivariate associations of severe intimate partner violence (IPV) with the stress of bride price and the stress of poverty

	Total respondents	Physical and severe psychological abuse ^b^	
		Row: % (n)	Adjusted OR (95%CI) ^c^
Ongoing poverty^a^			
No problem at all (ref. category)	802	18.2(146)	1.0
Some problem	667	31.2(208)	1.60 (1.23–2.07)*
Moderately serious/very serious problem	202	37.6(76)	1.75 (1.20–2.56)*
*p values from Chi-square test*		*p = 0.01*	
Problems with lia (bride price)			
No problem at all (ref. category)	899	18.0 (162)	1.0
A bit of a problem	600	32.2 (193)	1.85 (1.43–2.39)*
Moderately serious/very serious problem	172	43.6 (075)	2.73 (1.86–4.01)*
*p values from Chi-square test*		*p = 0.01*	

^a^Poverty included the following three items: not enough food; poor shelter; not enough money for school fees. Each item is scored 1 to 4: 1. not a problem, 2. a bit of problem, 3. a moderately serious problem, 4. a serious problem; total score range 3 to 12)

^b^Physical and severe psychological abuse includes any physical abuse (*n* = 103) and severe psychological abuse (threatening, intimidating and controlling behaviour) (*n* = 437)

^c^Adjusted OR: odds ratios are adjusted for age, educational status, occupation, and whether first or subsequent pregnancy.

Note: * Odds ratios are significant at *p*<0.05

A dose–response relationship was evident between the severity of problems with bride price stress and having experienced IPV. Those who reported moderate or serious problems with bride price stress had higher rates of IPV compared with those reporting no problems with bride price stress (43.6% vs. 18.0%, *p* < 0.01).

Multivariate analyses adjusting for socio-demographic and pregnancy-related factors, showed that bride price stress and poverty-related stress each made a statistical contribution to IPV; the highest adjusted odds ratio was for bride price stress (AOR = 2.73, 95%CI: 1.86–4.01) followed by poverty (AOR = 1.75, 95%CI: 1.20–2.56) (Table 3).

## Discussion

Our main findings were that a quarter of Timorese women in the antenatal period reported severe forms of IPV. Taking into account socio-demograhic and pregnancy related factors, the stress of bride price was the strongest predictor of IPV, followed by poverty. These associations remained strong and independent after controlling for a range of socio-demographic factors in the multivariate analysis. This is the first consecutively sampled study of pregnant women worldwide to demonstrate this finding.

Understanding of the socio-cultural complexities of bride price in Timor-Leste is assisted by our teams’ long engagement in the country, our prior exploratory research in this area, and our long association with Timorese research colleagues and advisors from the Alola Foundation, the peak non-government organization for women in that country [8]. A strength of the study is the sample size, which is the largest ever recruited for a study of IPV in a low-income, conflict-affected country. Recruitment covered all major antenatal clinics in a populous administrative district including the capital city. The consecutive approach to recruitment and the high response rate are additional strengths of the study. We applied the World Health Organisation measure for IPV, the standard approach used across multiple countries [30]. Limitations of the study are that it is cross-sectional in design, cautioning against drawing causal inferences regarding the relationships we found. In addition, we are not able to ascertain whether bride price stress or IPV were exacerbated or reduced during the pregnancy.

We found an association between younger age women and the stress of bride price. This association may be explained by the immediate duress of bride price obligations related to the wedding and ceremonial expenses.

In general, it is noteworthy that the overwhelming majority of the 836 million people recorded as experiencing extreme poverty in the contemporary world live in regions where bride price is a common practice, the most densely populated regions being in Southern Asia and sub-Saharan Africa [33]. As expected, in preliminary bivariate analyses, a strong association was found between the stresses of bride price and poverty. The cause of the association with poverty could be unidirectional or bidirectional. For example, the stress of paying bride price could be exacerbated by economic adversity, or the payment of the bride price could add further to the stresses of poverty; or both effects may operate simultaneously.

Directionality is more easily understood in relation to the path from bride price to IPV. The supposition is that gender inequality increases the risk of IPV [20, 21] and bride price contributes to gender inequality [17]. In this context our study supports a theory that through a process of traditionally bound inequality and the commodification women, the bride price symbolizes a gendered power imbalance and at the same time represents a concrete source of financial burden, the overall effect being to increase risk of IPV.

Our study therefore points strongly to the need for a focus on addressing the risk of bride price stress and reducing poverty in interventions designed to prevent or respond to IPV. Our study also suggests the need for bride price and poverty-related stressors to be considered in mental health and community development interventions.

A few studies and commentaries have associated financial stress and bride price with early marriage for girls and women in Africa and East Asia [34–37]. Early marriage has in turn been associated with poor maternal health outcomes as well as an increased risk of exploitation and violence against women who have less social power and are more vulnerable to abuse. In this context, the sustainable development agenda includes the goal to prevent early marriage [21]﻿. Our study adds the weight of empirical evidence to contend that global human rights and health theory should position bride price alongside early marriage and poverty as a key risk factor for IPV.

## Conclusion

Our findings, showing an association with IPV and bride price, add substantive evidence to the growing body of knowledge concerning the factors that increase risk of IPV. IPV represents a major risk factor for adverse physical and mental health outcomes amongst women in low income, conflict-affected countries [3]. Health is a basic human right, and good health enables women to engage fully in programs of poverty reduction essential to achieving the global sustainable development goals in which gender equality is a priority. In concrete terms, reduction of IPV and the elimination of early marriages are specific goals [20, 21]. In this context our findings suggest the need for a priority focus, in theory, research, policy and practice, on reducing the stress of bride price both to prevent the commodification of women and to curtail risk of IPV. These are essential elements in promoting women’s health and participation in development.

Bride price affects an estimated 2 billion women making it a potential global target in promoting women’s status and health. Our study offers an important contribution to the global development challenge to reduce IPV and improve women’s health in low-income and conflict-affected countries.

## Additional file

Additional file 1
**A:** Association between poverty-related stress and bride price stress. (DOCX 13 kb)
