# Supplementary material for: Associations between bride price stress and intimate partner violence amongst pregnant women in Timor-Leste
Source: Global Health. 2017 Aug 28;13:66. doi: 10.1186/s12992-017-0291-z (PMC5574248; doi:10.1186/s12992-017-0291-z)
Supplement: Additional file 1 — A: Association between poverty-related stress and bride price stress. (DOCX 13 kb) [file 12992_2017_291_MOESM1_ESM.docx]

**Additional File 1: A: Association between poverty-related stress and bride price stress.**

|  | Total number of respondents | **bride price stress:** Moderate/serious  /very serious problem |
| --- | --- | --- |
| Ongoing poverty-related stress | ***Number*** | Row % (n) |
| ***All*** | ***1672*** | ***10.3 (173)*** |
|  |  |  |
| **Lack of food:** Not a problem | 1396 | 7.4 (104) |
| A bit of problem | 239 | 21.8 (52) |
| Moderately serious problem | 25 | 36.0 (09) |
| A serious/very serious problem | 12 | 66.7 (08)) |
| *p values from Chi-square test* |  | *<0.001* |
|  |  |  |
| **Poor shelter:** Not a problem | 1238 | 7.7 (95) |
| A bit of problem | 361 | 15.2 (55)) |
| Moderately serious problem | 47 | 29.8 (14)) |
| A serious/very serious problem | 26 | 34.6 (09) |
| *p values from Chi-square test* |  | <0.001 |
|  |  |  |
| **Lack of money:** Not a problem | 1065 | 4.4 (47) |
| A bit of problem | 480 | 14.2 (68) |
| Moderately serious problem | 79 | 45.6 (36) |
| A serious/very serious problem | 48 | 45.8 (22) |
| *p values from Chi-square test* |  | <0.001 |
|  |  |  |
| ***Ongoing poverty*** |  |  |
| *No problem at all* | *802* | *3.5 (28)* |
| *Some problem* | *668* | *10.5 (70)* |
| *A serious/very serious problem* | *202* | *37.1 (75)* |
| *p values from Chi-square test* |  | <0.001 |
